# Principles of *in vitro* selection of ribozymes from random sequence libraries

**DOI:** 10.1098/rsif.2024.0878

**Published:** 2025-04-16

**Authors:** Paul G. Higgs, Ulrich F. Muller

**Affiliations:** ^1^ Department of Physics and Astronomy, Faculty of Science, McMaster University, Hamilton, Ontario, Canada; ^2^ Department of Chemistry, University of California San Diego, La Jolla, CA, USA

**Keywords:** ribozymes, *in vitro* selection, aptamers, RNA structure

## Abstract

*In vitro* selection methods are used to identify catalytic RNAs from pools of random sequences. We discuss the central concepts using experimental data and computational models. Experiments proceed in multiple rounds, each with a reaction step and a step in which reacted sequences are recovered. Sequences are enriched each round by a factor depending on combined reaction and recovery probability. In the first round, there are few functional sequences, and it is necessary to minimize the probability of losing these. In later rounds, the loss probability is negligible, and the procedure can be optimized to maximize the enrichment factor. Clusters of related sequences emerge which descend from separate sequences in the initial pool. The fitness of an RNA depends on how well it matches a structure with specified sequence and base-pair constraints. Sequences that exactly match the constraints may be rare, but sequences a few mutations away are much more common; hence it is likely that clusters descend from suboptimal sequences. There is a high probability that beneficial mutations arise during the experiment. This explains the experimental observation that there is little correlation between cluster frequencies and fitnesses, whereas correlation between enrichment factors and fitnesses is strong.

## 1. Introduction

The selection of functional sequences from libraries of random sequences was invented around 1990 [1,2]. The term ‘SELEX’ (systematic enrichment of ligands by exponential amplification [2]) is used for RNA ‘aptamers’ that tightly bind to ligands [1], while the term ‘*in vitro* selection’ is used more generally for RNA aptamers as well as for ‘ribozymes’ that catalyse chemical reactions [3,4]. Importantly, the method does not require prior knowledge of *how* an RNA may function. The many sequences in an RNA library will fold to complicated three-dimensional (3D) structures, which in rare cases will be just right to bind a given ligand, or catalyse a given reaction.

In this article, we give a theoretical model that is intended to illustrate the way this method works and to show some of the common factors that are likely to arise in many experiments of this type. Before describing the model, however, we wish to give examples of how *in vitro* selection has been used, and to describe the important questions that a theoretical model must address.

In the first approximately 15 years of *in vitro* selections, a central question was ‘which reactions can be catalysed by RNA?’. During this phase, ribozymes were developed that catalysed RNA–RNA ligation [3], kinase reactions [5], alkylation [6], peptide bond formation [7], N-glycosidic bond formation in nucleotides [8], Diels–Alder reactions [9], RNA polymerization [10], alcohol reduction [11], aldol reactions [12] and others, showing that most reactions catalysed by enzymes can also be catalysed by ribozymes. These studies helped to understand naturally coccurring ribozymes, including self-cleaving hammerhead ribozymes [13], self-splicing introns [4,14], the spliceosome [15], RNase P [16] and the ribosome [17]. Artificial selection found vastly more ribozymes than in nature, and identified hundreds of different RNAs catalysing the same reaction (e.g. [18]) where in nature there had been none or only a few. Many ribozymes were selected with the goal of testing how an RNA-dominated, early stage of life could have replicated and functioned [10,19–21]. The first aptamers were selected to bind to blue dyes [1], and later studies found that aptamers are able to discriminate between substrates that differ by as little as one methyl group [22]. The affinity and specificity of RNA aptamers have been used in microscopy, with dye-binding aptamers that generate an excellent signal-to-noise ratio [23], and to manage blood coagulation [24] in clinical settings. These results show that *in vitro* selection of RNAs has increased our insight into many biological processes, possible origins of life, and generated useful tools for applications in research and clinic.

An *in vitro* selection experiment involves reaction, recovery and amplification steps, which are repeated over multiple rounds. In the reaction step, sequences are allowed to react for a time during which the desired reaction occurs. In the recovery step, there is a means of recovering sequences that have reacted from the mixture of reacted and unreacted sequences. In the amplification step, the recovered sequences undergo several cycles of PCR so that a sufficiently large number of sequences can be input to the next round of the procedure.

**Figure 1.**
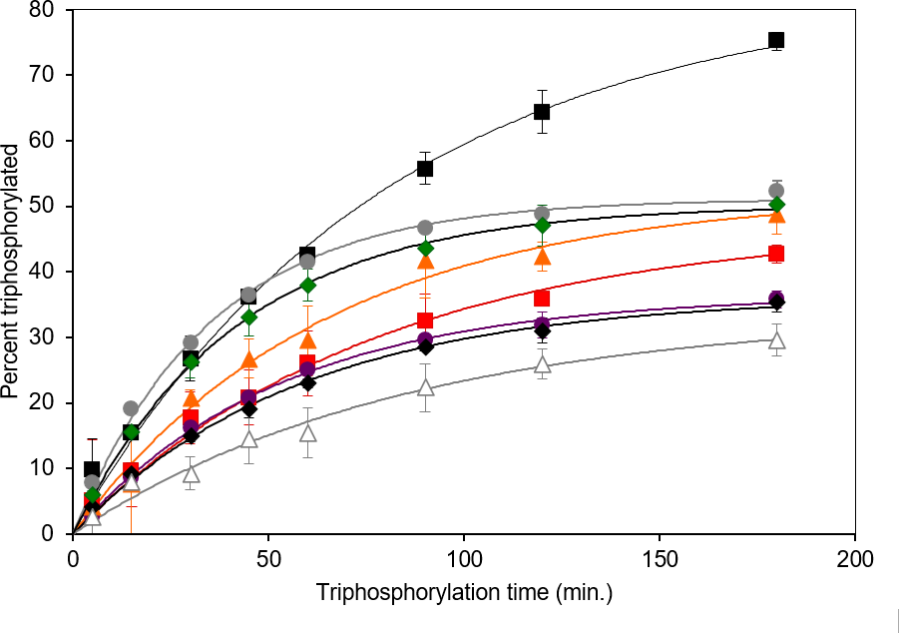
Reaction kinetics of ribozymes that were initial isolates from an *in vitro* selection procedure. Reproduced from [25]. Shown is the percentage of ribozymes having completed a self-triphosphorylation reaction as a function of reaction time. Each curve corresponds to a different RNA sequence that was isolated in the same selection experiment.

For a first-order reaction, the probability that a catalytic sequence has reacted after time t is $r_{cat}\left(t\right)=A(1-exp(-k_{cat}C_{sub}t))$, where $k_{cat}$ is the rate constant and $C_{sub}$ is the substrate concentration, e.g. for self-triphosphorylating ribozymes, the substrate is trimetaphosphate, which reacts with the ribozyme’s 5′-hydroxyl to form 5′-triphosphates. For self-cleaving ribozymes like the hammerhead ribozyme, there is no substrate, and $C_{sub}$ is simply 1. The amplitude A denotes the maximum fraction of sequences that react after a long time. Figure 1 shows examples of self-triphosphorylating ribozymes [25], where it was found that A varied in the range 0.3−0.8. The reason A is less than 1 is probably that a significant proportion of sequences become trapped in unreactive misfolded configurations. If a sequence misfolds, it often cannot refold to a functional structure during the reaction time of the experiment.

The majority of random sequences are not catalysts, but they may undergo the reaction at a slow, uncatalysed, ‘background’ rate, $k_{back}$. For example, in the case of self-triphosphorylating ribozymes, there is some background rate of phosphorylation at the 5′ end of a sequence in the absence of any particular catalytic structure of the sequence. The efficiency with which the selection procedure amplifies catalytic sequences depends on the ratio $k_{cat}/k_{back}$. Ribozymes will be difficult to isolate if this ratio is too low.

The rate at which catalytic sequences are amplified depends on the stringency of selection. It is necessary to have low stringency in early rounds to allow time for reactive sequences to complete the reaction so they are captured efficiently from the initial mixture, and not lost due to statistical effects that are important when each sequence is represented only by one, or a handful of molecules. In later rounds, it is desirable to increase the stringency of selection to give greater discrimination between optimal and suboptimal catalysts. This can be done by decreasing the reaction time, decreasing substrate concentration, or both. For a one-step reaction, halving the reaction time is equivalent to halving the substrate concentration. As examples, neither reaction time nor substrate concentrations were adjusted in [7,26], the reaction time was adjusted in [3,12,25,27–29], the substrate concentration was adjusted in [30], and both reaction time and substrate concentration were adjusted in [8,31–33].

In the recovery step, Q is the probability that a reacted sequence is recovered from the mixture of reacted and unreacted sequences. Q is affected by inefficiencies in standard processing steps that are necessary in the procedure, such as incomplete enzymatic processing or losses during gel purification. Hence Q may be significantly less than 1, and we use Q=0.2 for the examples in this paper.

**Figure 2.**
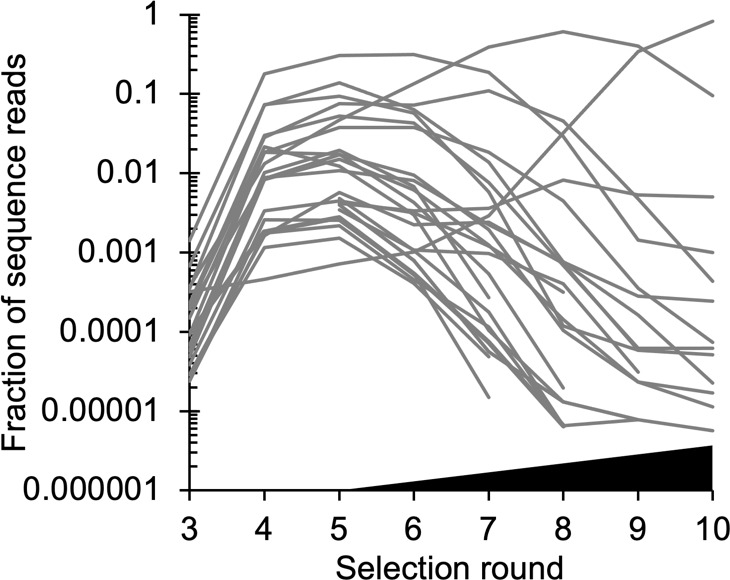
Frequencies of clusters of catalytic sequences for self-triphosphorylating ribozymes (reproduced from [33]).

The progress of an experiment can be followed by sequencing strands from the mixture. These sequences can be clustered into groups with related nucleotide sequences. Clusters exist because the random mixture that enters the first round of selection contains a fairly small number of functional molecules that are very different from each other. Imperfect copying during the PCR stage creates clusters of similar sequences that derived from a single sequence in the original mixture. Figure 2 shows frequencies of sequence clusters in an experiment with self-triphosphorylating ribozymes [33].

Cluster frequencies increase exponentially at first because these sequences have higher fitness than background sequences. In later rounds, background sequences have been eliminated, and competition between clusters causes clusters of less than average fitness to decrease. In figure 2, there is some crossing over of the cluster frequencies. The cluster that increases most rapidly in the first few rounds is not necessarily the cluster that ends up with the highest frequency. This means that the fitnesses of clusters do not remain constant. This occurs either because the stringency of selection is increased or because beneficial mutations arise during the experiment.

From these preliminaries, we identify several general questions.

— How many random sequences are needed to give a good chance of isolating functional sequences?
— What factors determine the enrichment factor of functional sequences, and how can this be optimized?
— How rare are functional ribozymes in the random mixture?
— How likely is it that beneficial mutations occur during the experiment?
— Why is cluster fitness not strongly correlated with cluster frequency?

We aim to give a simple general theory that addresses these questions, which should be relevant to understanding previous results, and to designing efficient experiments in the future.

## 2. Results

### 2.1. The first round of selection

The experiment begins with a library of random DNA sequences. A small fraction of these sequences is functional. If sampling of sequence space is sparse, we expect that each functional sequence is only present in one copy. These sequences are typically amplified by a few cycles of PCR, and then RNAs are transcribed from the DNAs. If the number of DNA sequences prior to PCR is $N_{DNA}$ and the number of RNAs after PCR and transcription is $N_{RNA}$, then the mean number of copies of each of the functional sequences in the original library is ${c=N}_{RNA}/N_{DNA}$. How big must c be to make sure that most of the functional DNAs in the library are successfully amplified? The probability that at least one copy of any one sequence enters the first round is $1-{\left(1-1/N_{DNA}\right)}^{N_{RNA}}\approx 1-exp\left(-c\right)$. To ensure that at least 95% of the functional sequences enter the first round, we require c>−ln(0.05)≈3.

In practice, however, considerably more than three RNA copies are generated per DNA sequence because it is necessary to account for the loss of functional sequences during the first round. The probability of successful reaction and recovery is $R=Qr_{cat}(t)$, which is QA when t is long, as should be the case in the first round. If c copies enter the first round, the mean number of copies recovered is QAc. The probability that at least one copy is recovered is $1-{\left(1-QA/N_{DNA}\right)}^{N_{RNA}}=1-exp\left(-QAc\right)$. Hence, we now need QAc>3 to ensure that 95% of functional sequences are recovered. If A=0.5 and Q=0.2, so that QA=0.1, we need c>30. For example, in [21], the DNA library contained approximately $1.6\times 10^{14}$ sequences, and a total of $5.3\times 10^{15}$ RNA molecules entered the first round, which corresponds to an average copy number of c=33 for each RNA molecule.

### 2.2. Enrichment factors

We now consider a population containing catalysts labelled i=1,2,3… with rates and amplitudes $k_{i}$ and $A_{i}$. Let $k_{cat}$ be the rate for the best catalyst in the population. We define a scaled time $\tau =k_{cat}C_{sub}t$, such that the mean time for the best catalyst to complete the reaction is τ=1. The rest are background sequences with rate $k_{back}\ll$$k_{cat}$. The recovery probability Q is the same for all reacted sequences, whether background or catalyst. The combined probabilities of reaction and recovery are


$$R_{i}\left(\tau \right)=QA_{i}\left(1-\exp\left(-\tau k_{i}/k_{cat}\right)\right),$$

$$R_{back}\left(\tau \right)=Q\left(1-\exp\left(-\tau k_{back}/k_{cat}\right)\right).$$

We assume A=1 for a background sequence, because the background reaction is usually independent of structure. An artefact is a sequence that is recovered from the separation step without catalysing the reaction. An example would be a sequence that binds with high affinity to the agarose matrix in a separation step where the intended product of the ribozyme reaction is coupled to the agarose matrix. The probability of recovery of an artefact is $R_{art}$, which depends on the separation method but which is independent of the reaction time τ.

Let $p_{i}\left(n\right),\;p_{art}\left(n\right)$ and $p_{back}(n)$ be the frequencies of the sequences after selection round n, and let $p_{i}(n-1),p_{art}(n-1)$ and $p_{back}(n-1)$ be the frequencies before round n (i.e. after round n-1). The mean probability of reaction and recovery during round n is


$$\bar{R}=p_{back}\left(n-1\right)R_{back}\left(\tau \right)+p_{art}\left(n-1\right)R_{art}+\sum_{i}p_{i}\left(n-1\right)R_{i}\left(\tau \right).$$

The frequencies after round n are


$$p_{i}\left(n\right)=p_{i}\left(n-1\right)R_{i}\left(\tau \right)/\bar{R},$$

$$p_{art}\left(n\right)=p_{art}\left(n-1\right)R_{art}/\bar{R},$$

$$p_{back}\left(n\right)=p_{back}\left(n-1\right)R_{back}\left(\tau \right)/\bar{R}.$$

The enrichment factors for catalysts and artefact are


$$E_{i}=p_{i}\left(n\right)/p_{i}\left(n-1\right)=R_{i}\left(\tau \right)/\bar{R},$$

$$E_{art}=p_{art}\left(n\right)/p_{art}\left(n-1\right)=R_{art}/\bar{R}.$$

In the first few rounds, catalysts and artefacts are rare; therefore $\bar{R}=R_{back}\left(\tau \right)$. For the best catalyst with $k_{i}=k_{cat}$, the enrichment factor in the first few rounds is


$$E_{cat}=\frac{R_{cat}\left(\tau \right)}{R_{back}\left(\tau \right)}=\frac{A_{i}\left(1-\exp\left(-\tau \right)\right)}{1-\exp\left(-\frac{\tau k_{back}}{k_{cat}}\right)},$$

and for the artefact,


$$E_{art}=\frac{R_{art}}{R_{back}\left(\tau \right)}=\frac{R_{art}}{Q\left(1-\exp\left(-\frac{\tau k_{back}}{k_{cat}}\right)\right)}.$$

**Figure 3.**
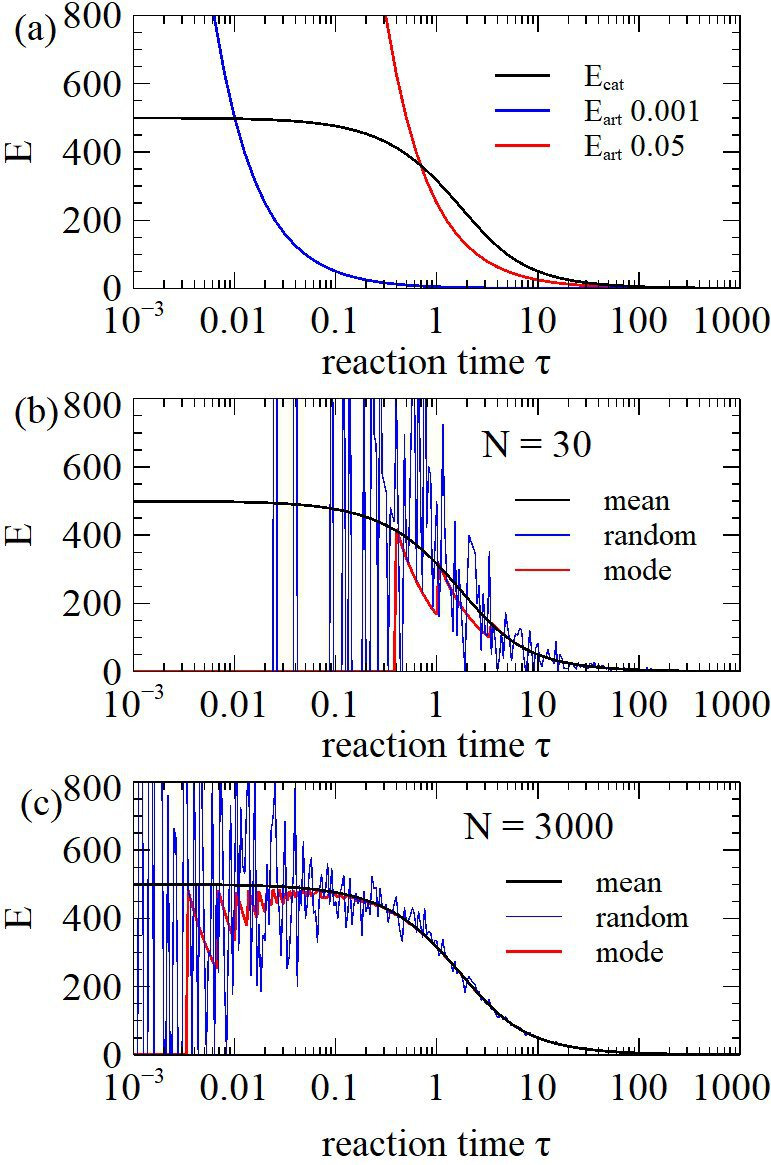
(a) Probability R of recovery of at least one copy of a functional sequence as a function of reaction time, given that the number of sequences that enter the selection round is either N = 30 or 3000. (b) Enrichment factor when N=30 calculated using the mean number of recovered sequences $\bar{N_{rec}}$, or using a random value of $N_{rec}$ from the binomial distribution, or by setting $N_{rec}$ to the mode of the distribution. (c) As (b) except N=3000.

The maximum attainable $E_{cat}$$=Ak_{cat}/k_{back}$, when τ≪1. If τ is increased, a larger fraction of background sequences reacts, so $E_{cat}$ decreases. This is shown in figure 3a for a case with $k_{cat}/k_{back}$ = 1000 and $A_{i}=0.5$. $E_{cat}$ approaches 500 for τ≪1 and decreases significantly for τ>1. The figure also shows $E_{art}$ for artefacts with two different recovery probabilities. If there were an artefact with $R_{art}$ = 0.001 it would be possible to operate with τ=0.1, and the enrichment factor for the catalyst would be close to the maximum value of 500. If there were an artefact with $R_{art}=0.05$, it would be necessary to have τ§amp;gt;1 in order to prevent amplification of the artefact, and the enrichment factor of the catalyst would be only about half the maximum value. This makes it clear that the separation step should be designed to keep $R_{art}$ as low as possible. In reality, the rate parameters of desired sequences are not known; therefore the reaction time needs to be considered based on the catalysed and uncatalysed rates of similar reactions, and the estimated frequency of artefacts.

The enrichment factor calculated above ignores fluctuations arising from small copy numbers. If N copies of the catalyst enter a selection round the total number of sequences is $N_{tot}$, the frequency before the round is $p(n-1)=N/N_{tot}$. The probability that $N_{rec}$ copies are recovered is


$$P\left(N_{rec}\right)={\left(R_{cat}\left(\tau \right)\right)}^{N_{rec}}{\left(1-R_{cat}\left(\tau \right)\right)}^{N-N_{rec}}\frac{N!}{N_{rec}!\left(N-N_{rec}\right)!},$$

i.e. the actual number of catalysts recovered is $N_{rec}$, chosen from this calculated with the mbinomial distribution. The total number of sequences recovered is $N_{tot}R_{back}(\tau )$ (as almost all sequences are background initially). Therefore, the frequency of the sequence after one round is $p(n)=N_{rec}/N_{tot}R_{back}(\tau )$ and the enrichment factor is $E=p(n)/p(n-1)=N_{rec}/NR_{back}(\tau )$. The mean value of $N_{rec}$ is $\bar{N_{rec}}=NR_{cat}\left(\tau \right)$. If we set $N_{rec}=\bar{N_{rec}}$, then $E=R_{cat}(\tau )/R_{back}(\tau )$, as above. However, when $N_{rec}$ is small, the mean does not give a good prediction of the outcome. Figure 3b compares E calculated with the mean $\bar{N_{rec}}$ with E obtained from a random $N_{rec}$ chosen independently from the binomial distribution for each value of τ. If N=30, there are large fluctuations. The most likely value of $N_{rec}$ is the mode (the $N_{rec}$ for which $P(N_{rec})$ is largest). Figure 3b also shows the enrichment factor if $N_{rec}$ is set to the mode. The mode is NQA=3 when τ is large, but the mode falls to zero when τ is small, so the most likely outcome is to lose the sequence altogether, even though the mean remains high. Hence, we need to keep τ>1 in the first round when N=30. Figure 3c shows the same things with 𝑁 = 3000. Fluctuations become important only at much lower values of τ. If we work with τ around 0.1, the mean is still a good indication of the outcome, and the chance of loss of the sequence is low. As the number of sequence copies increases usually more than 10-fold in each round of selection, the fluctuations considered here become insignificant after one or two rounds.

In summary, in the first round, avoiding loss of functional sequences is the most important factor that limits the rate of enrichment, whereas in subsequent rounds, the most important factors are the ratio of catalytic rates $k_{cat}/k_{back}$ and the avoidance of artefacts.

We now consider examples of enrichment factors from real experiments. This factor can be estimated from the initial size of the library and the number of rounds until active sequences dominate. Most enrichment factors are between 20-fold and 1000-fold. Specifically enrichment factors were around 20-fold [26,32], 30-fold [8], 50-fold [7], 60-fold [22], 80-fold [12], 240-fold [9] and 1100-fold [28]. The two highest enrichment factors were 5700-fold [27] and around 10 000-fold [25]. The latter resulted from a low background reaction rate of 1.4 × 10^–8^ min^–1^, and catalysed rates around 0.02 min^–1^, which gives a maximum possible E=7.1 × 10^5^ if A=0.5 [25]. However, this maximum E was suppressed to about 10 000 to avoid PCR artefacts. This high E was confirmed in several studies that used the same selection system [33,34]. The lowest enrichment factor of about 12-fold in a different study resulted from the relatively high background reaction rate [21]. A low enrichment factor makes it more likely for artefacts to arise, which did occur in that study, and required a modification in the selection step to avoid the artefact. These examples confirm that high enrichment factors are preferable but that enrichment factors down to 12-fold have been used successfully in real cases.

### 2.3. Selection of catalysts over multiple rounds

As an example showing how sequences are selected over multiple rounds, we consider a sequence pool containing catalytic sequences 1−4 with rates and amplitudes shown in table 1. The recovery probability is Q=0.2 for all sequences that react.

**Table 1.** Properties of the example sequences.

sequence i	$A_{i}$	$k_{i}$	$A_{i}k_{i}$
1	0.55	1.0	0.55
2	0.5	0.8	0.4
3	0.7	0.6	0.42
4	0.5	0.5	0.25
background	1.0	0.001	0.001

**Figure 4.**
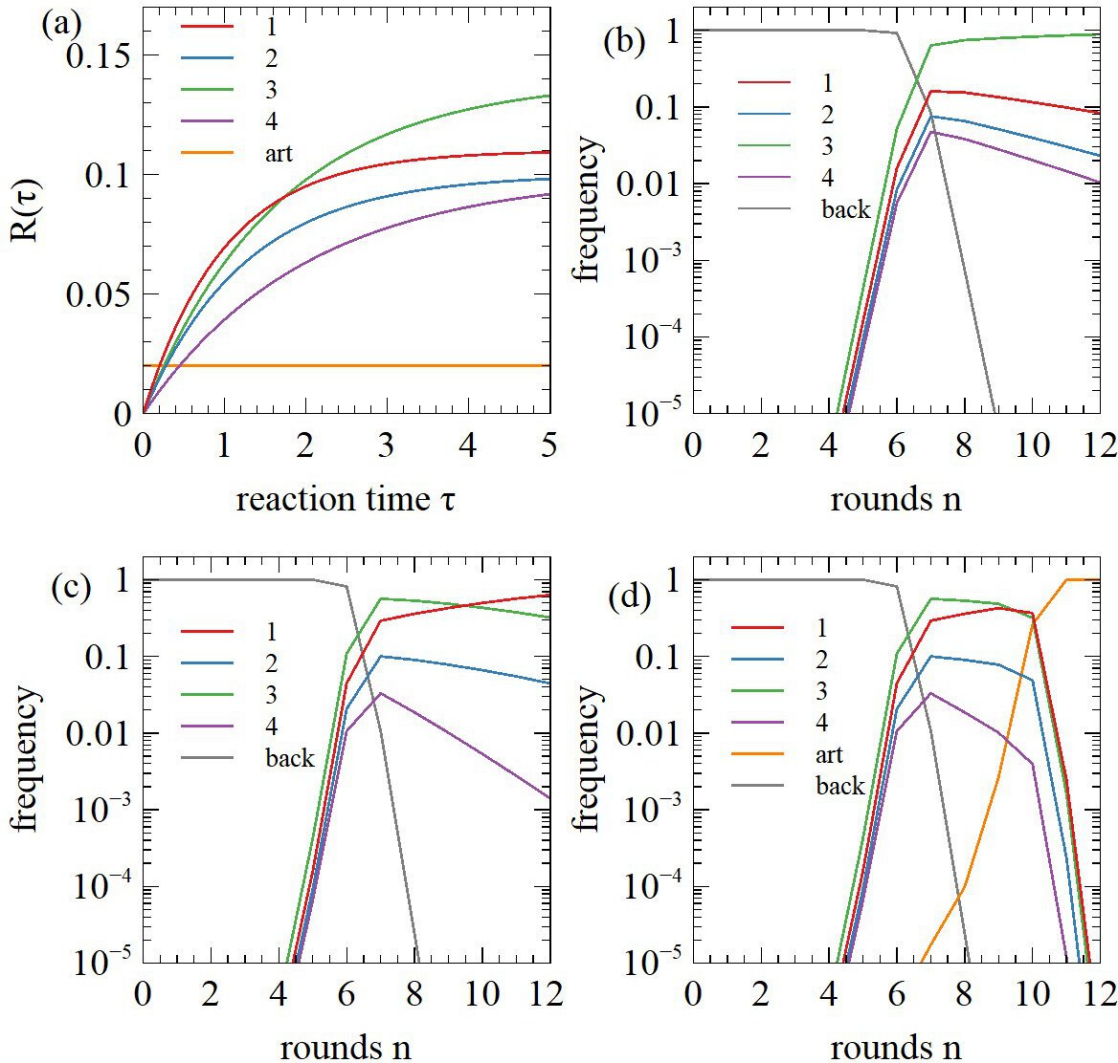
(a) Probability $R_{i}(\tau )$ for reaction and recovery of four catalytic sequences as a function of reaction time τ in comparison with the recovery probability of an artefact with $R_{art}=0.02$. (b) Frequencies of the catalysts and the background sequences with reaction time fixed at τ=5. (c) As for (b), but τ is decreased in later rounds. (d) As for (c), but the artefact is present. The artefact takes over the population when τ becomes too small.

Figure 4a shows the reaction and recovery probabilities $R_{i}(\tau )$ for these sequences. When τ=5 all reaction curves are approaching saturation. The ranking of the sequences is 3>1>2>4, which is mainly determined by $A_{i}$. For small τ the ranking is 1>3>2>4, which is determined by the product $A_{i}k_{i}$. Additionally, we consider an artefact with recovery probability $Q_{art}=0.02$. For small enough τ the artefact has a higher recovery probability than any of the catalysts.

The initial frequencies are $p_{i}\left(0\right)=10^{-14}$. Figure 4b shows how these frequencies change over selection rounds. We set τ=5 to achieve a high reaction and recovery probability of functional sequences. There is no artefact in this case. The frequency of the catalysts increases exponentially and becomes visible on this scale after four rounds. After about eight rounds the background sequences are eliminated and the catalysts compete against each other. Sequence 3 is dominating because it has the largest $A_{i}$.

In figure 4c, the stringency of selection was increased in later rounds. We set τ=5 in rounds 1−5, and progressively decrease τ by a factor of 5 every round from round 6 onwards. When τ is small, $r_{i}(\tau )\approx A_{i}k_{i}\tau /k_{cat}$. Sequence 1 becomes most frequent, because it has the highest value of $A_{i}k_{i}$. If τ is constant over selection rounds, the frequency curves for the four different catalysts never cross. If τ is decreased in later rounds, the frequency curves can cross (as in figure 4c). This is one possible explanation of the observed crossing of frequencies in figure 2.

We now consider artefacts. In figure 4d, the artefact is included with initial frequency $p_{art}\left(0\right)=10^{-14}$ and the reaction time is decreased in later rounds as in figure 4c. The artefact frequency is negligible in the early rounds, but when τ is decreased, $R_{art}$ becomes higher than $R_{i}(\tau )$ for the fastest catalyst, so the artefact eliminates the catalysts. If an artefact like this appears, it may be possible to remove it by changing the conditions of the separation step. For example, in [21], active sequences were captured during aminophenyl mercury–polyacrylamide gel electrophoresis at the top of the mercury layer in the gel. An artefact appeared at the same position in the gel (fig. S1C of [21]), probably due to dimer formation. It was possible to remove the artefact by increasing the time of electrophoresis such that the slowly moving sequences were removed from the position where active sequences were isolated.

### 2.4. Conditional ribozyme function and counter-selection methods

Many researchers have used *in vitro* selection to select for ribozymes whose function is conditional on the presence of a particular cofactor molecule. Examples include allosterically regulated self-cleaving ribozymes [35–38], sensors for caffeine [39], protein-dependent ribozymes [40] and promoter-dependent RNA polymerases [41]. In these experiments, it is desired to find a ribozyme that works in the presence of the cofactor but not in its absence. For this reason, a counter-selection step can be added to select against sequences that perform the reaction without using the cofactor. In each round of selection, reaction and recovery steps are first performed in the absence of the cofactor. Sequences that are successful at this stage are discarded. The remaining sequences are put through a second reaction and recovery step in the presence of the cofactor, and sequences that are successful in the second step are retained to the next round. The order of these two steps can be changed but that does not change the considerations below. We now consider under what conditions we would expect a counter-selection process to be successful.

We consider an example with three catalytic sequences. Sequence 1 functions without binding to the cofactor. It has rate and amplitude parameters $k_{1}$ and $A_{1}$ which are the same with and without the cofactor. Sequence 2 is a desired sequence: it binds to the cofactor and has parameters $k_{2}$ and $A_{2}$ with the cofactor, but without the cofactor it is not active and behaves in the same way as a background sequence. Sequence 3 binds to the cofactor and functions well in the presence of the cofactor, but also functions to a lesser extent without the cofactor. It has parameters $k_{3}$ and $A_{3}$ with the cofactor, but reduced rate $k_{3}/2$ without the cofactor.

**Figure 5.**
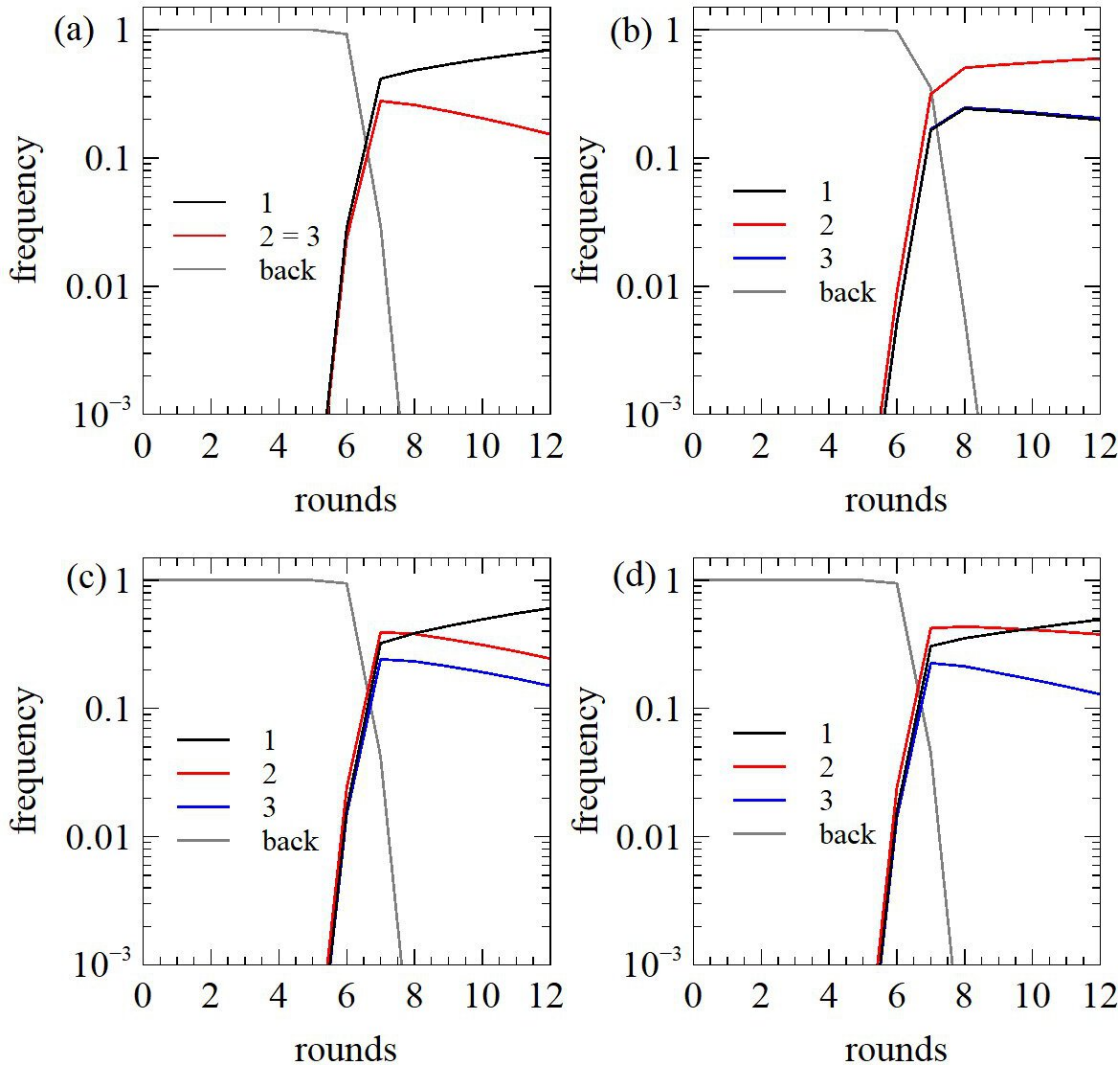
Selection of a ribozyme that functions conditionally on the presence of a cofactor. The model compares the behaviour of sequences that are constitutionally active (sequence 1, black), the desired sequence 2 that is active dependent on the cofactor (red), and a sequence that depends on the cofactor for high activity but has some activity without cofactor (sequence 3, blue). Background sequences without activity are shown in grey. (a) Direct selection in the presence of the cofactor with τ decreasing in later rounds. (b) Counter-selection plus direct selection, both with constant τ. (c) Counter-selection with direct selection, both with decreasing τ. (d) Counter-selection at fixed τ, plus direct selection with decreasing τ.

As an example, we choose $k_{1}=1,k_{2}=0.8,k_{3}=0.8$ and $A_{1}=A_{2}=A_{3}=0.5$. Background sequences have rate $k_{back}=0.001$ and amplitude A=1 as before. We define the scale time relative to the rate of sequence 1: $\tau =k_{1}C_{sub}t$. If the standard direct selection method is used with the cofactor present, the sequence with the highest k is selected (because the amplitudes are equal). This is sequence 1 in this case (shown in figure 5a). We have used τ=5 for the first five rounds and then reduced τ by a factor of 5 each round from round 6 onwards. If $k_{2}$ were the highest, then sequence 2 would be selected by direct selection, in which case counter-selection would not be needed.

When counter-selection is performed, the probability of passing through to the next round is the probability of not being recovered without the cofactor multiplied by the probability of being recovered with the cofactor. The probabilities of reaching the next round are


$$R_{1}(\tau )=(1-QA_{1}(1-exp(-\tau ))QA_{i}(1-\exp(-\tau )),$$

$$R_{2}(\tau )=(1-Q(1-\exp\;(-\tau k_{back}/k_{1}))QA_{2}(1-\exp(-\tau k_{2}/k_{1})),$$

$$R_{3}(\tau )=(1-QA_{3}(1-exp(-\tau k_{3}/2k_{1}))QA_{3}(1-\exp(-\tau k_{3}/k_{1})),$$

$$R_{back}(\tau )=(1-Q(1-exp\;(-\tau k_{back}/k_{1}))\;Q\;(1-\exp(-\tau k_{back}/k_{1}t)).$$

Figure 5b shows that sequence 2 can be selected when counter-selection is performed, even though it has a lower k. In this example τ was kept constant at 5. If τ is reduced in later rounds, the counter-selection does not work. In figure 5c, τ was reduced in later rounds in both the counter-selection step and the positive selection step. In this case, sequence 1 is selected. Counter-selection relies on removal of the sequences which react in the absence of the cofactor. Therefore if τ is decreased in the counter-selection step, it will be less effective, because fewer sequences will be removed. However it is not necessary to have the same τ in the counter-selection and positive selections steps. We tried an example where τ was fixed for the counter-selection step but decreased in later rounds for the positive selection step. Again this did not work—sequence 1 was selected (figure 5d). In this case, the selection against sequence 1 was maintained in the counter-selection step, but the selection in favour of sequence 1 was increased in the positive selection step, and the latter was most important. It should be remembered that since Q and A are less than 1, only a fraction of reactive sequences actually perform the reaction, and those that do not react will not be removed by the counter-selection step, so at best counter-selection is fairly weak, and is sometimes overcome by the positive selection step.

Before leaving the topic of ribozymes whose function is conditional on the presence of a cofactor, we note that a different method to identify such RNAs has become available through the use of high throughput sequencing. Here, the number of sequence reads are compared between two parallel selection rounds, where one line is selected in the absence of the compound, the other in the presence of the compound [33].

### 2.5. Frequency of sequences that match specified RNA structures

**Figure 6.**
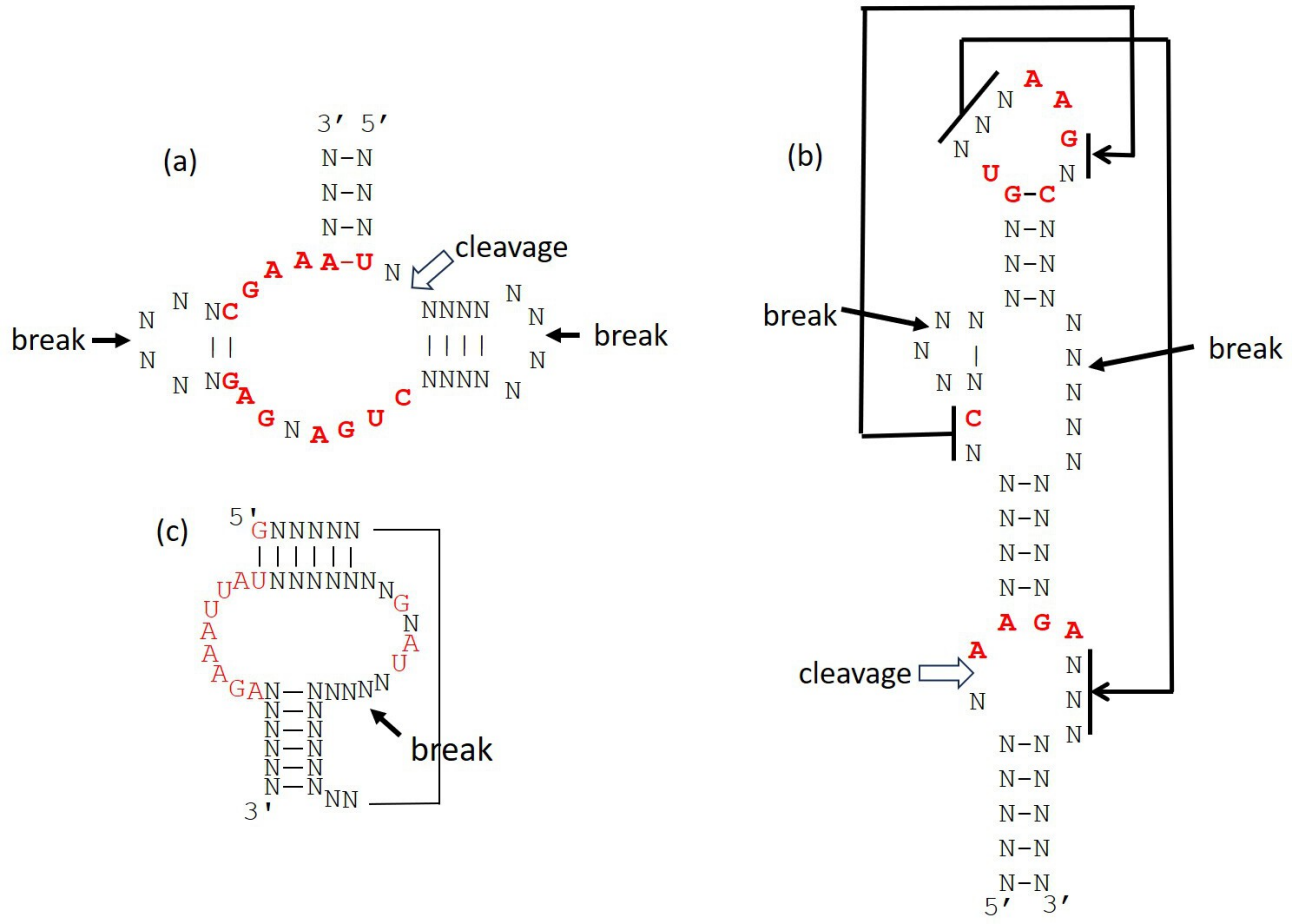
Conserved secondary structures of three ribozymes. Conserved bases are shown in red. *N* indicates variable sites. Paired variable sites *N*–*N* must satisfy base pairing constraints. (a) Hammerhead self-cleaving ribozyme [42], (b) twister self-cleaving ribozymes [42,43], (c) the smallest known self-triphosphorylation ribozyme [44]. Large arrows indicate cleavage sites. Smaller arrows indicate break points where additional bases can be inserted with only minor effect on the function.

The selection principles discussed above do not depend on the structure of the catalyst. However, the likelihood of obtaining a functional catalyst depends on the structure. For several ribozymes, mutational studies have determined the structural constraints. Figure 6 shows three examples: the hammerhead and twister self-cleaving ribozymes and a self-triphosphorylation ribozyme.

Certain positions in the structure require specific nucleotides for function, which are conserved in almost all selected sequences that fit these structures. These positions are indicated in red. Other positions are variable, and are indicated by *N*. Additionally, the structures contain paired regions that must satisfy base pairing constraints. Each pair *N*–*N* must be GC, CG, AU, UA, GU or UG. The length of the randomized sequence is $L_{ran}$ and the length of the ribozyme structure is $L_{str}$. If $L_{ran}=L_{str}$, there is only one way in which the structure can be fitted into the randomized sequence. The frequency of sequences that match a structure depends on the number of conserved sites, s, and the number of base pairs, b. If the four bases have equal frequency, the probability of matching the base at a conserved site is ¼ and the probability that a canonical pair appears at a paired position is 3/8. The probability that a random sequence matches all the required constraints is $q={\left(1/4\right)}^{s}{\left(3/8\right)}^{b}$. Table 2 shows $L_{str},\;s,\;b$ and q for the three ribozymes in figure 6. Where conserved bases occur in a stem region, this is treated as two single-site constraints, and it does not count as a pairing constraint because the sequence constraints already enforce base pairing. Structures (*b*) and (*c*) contain pseudoknots, but a base pair constraint in a pseudoknot is the same as a base pair constraint in an ordinary stem of the secondary structure.

**Table 2.** Parameters for three example ribozymes.

	$L_{str}$	s	b	q	$L_{ran}$	M	W	Wq
hammerhead	39	13	8	$5.8\times 10^{-12}$	80	3	13 244	$7.7\times 10^{-8}$
						1	42	$2.4\times 10^{-10}$
twister	54	11	17	$1.4\times 10^{-14}$	80	3	3654	$5.0\times 10^{-11}$
triphosphorylation ribozyme	44	13	11	$3.1\times 10^{-13}$	80	1	37	$1.1\times 10^{-11}$

The probability q gives a rough estimate of the frequency of random sequences that match a given structure, but it does not account for the thermodynamic stability of a structure or for competing structures that are formed by the same sequence. Nevertheless, from this simple estimate it is clear that the frequencies of even fairly simple structures are quite small, and that structures differ in frequency considerably according to the number of conserved sites and base pair constraints.

We assumed so far that $L_{ran}=L_{str}$. However, in practice we do not know what the ribozyme structure is prior to doing the experiment, and any structure for which $L_{str}\leq L_{ran}$ could arise. If the structure occurs as a continuous sequence of $L_{str}$ bases, the number of ways of sliding the structure within the length $L_{ran}$ is $W=L_{ran}-L_{str}+1$. We will refer to W as the number of ‘frames’ in which the structure can be positioned. In addition to simply sliding the structure within the randomized section, many ribozymes have break points at which additional bases can be inserted without significantly affecting the function. Two break points are shown in the hammerhead structure in figure 6a. This divides the ribozyme into three modules, which can be moved separately. The number of sites that are not part of the structure is $L_{ran}-L_{str}$ and the number of modules is M. The number of permutations of $L_{ran}-L_{str}+M$ objects is $W=\left(L_{ran}-L_{str}+M\right)!/\left(L_{ran}-L_{str}\right)!M!$ . The probability of matching the structure in any one of these frames is q. If we treat these frames as independent, the probability of a match in at least one frame is $P_{W}=1-{\left(1-q\right)}^{W}\approx Wq$. This result was given by Sabeti *et al*. [45]. As $L_{ran}$ increases, W increases dramatically. This means there is a large advantage in having a longer randomized length $L_{ran}$, especially if the structure is divisible into more than one module.

Values of W are shown in table 2 for the three example ribozymes, assuming L=80. For both the hammerhead and twister ribozymes there are two break points, so M=3. For the hammerhead, we also show what would happen if there were no break points but the whole ribozyme could slide within the randomized length (i.e. if M=1). It is much more likely to find matching sequences if M=3 than if M=1. In the triphosphorylation ribozyme there is one break point, which gives two modules. However, triphosphorylation occurs at the 5′ end of the sequence, so the first module has to be at the 5′ end of the RNA. Only the second module is movable; hence the W factor is calculated with M=1, not M=2, in this case. For this reason, the final probability of finding the ribozyme, Wq, is less for the triphosphorylation ribozyme than for twister, even though q is smaller for twister.

Although larger $L_{ran}$ increases W and makes the ribozyme easier to find, sequences inserted at the break points, and sequence extensions at the 5′- or 3′-ends can interfere with ribozyme folding. However, as discussed by Sabeti *et al*. [45], this effect is usually less than one order of magnitude, while the benefits of containing the same secondary structure in different frames increase very rapidly with the length of the library. Thus, there is still a substantial advantage of larger $L_{ran}$, although the W factor probably overestimates this advantage.

In practice the library length is ≤200 nucleotides in most cases, otherwise the synthesis of a library with high complexity requires significantly more effort. Additionally, the PCR amplification is more efficient with amplicons ≤100 nucleotides, and the yield of RNAs from polyacrylamide gels decreases with increasing length. As a compromise between the combinatorial advantage, and technical difficulties with longer libraries, researchers have usually used libraries with random regions in the range 70 to 150 nucleotides [8,9,11,25,46].

### 2.6. Frequency of sequences at mutational distance *m* from a specified structure

The previous section considered the frequency of sequences that exactly match a set of structural constrains, but it is likely that an exact match not absolutely necessary, and that sequences within a few mutations also have a catalytic rate above the background rate. Furthermore, sequences that differ by a few mutations are much more frequent than sequences with an exact match. Therefore, we wish to estimate the frequency of sequences that are some distance away from an exact match.

Of the s conserved sites, let $m_{1}$ be the number of sites that do not match the required base, and of the b base pairs, let $m_{2}$ be the number of pairs at which the bases do not match. The frequency of such sequences is


$$P\left(m_{1},m_{2}\right)={\left(\frac{3}{4}\right)}^{m_{1}}{\left(\frac{1}{4}\right)}^{s-m_{1}}\frac{s!}{m_{1}!\left(s-m_{1}\right)!}\;{\left(\frac{5}{8}\right)}^{m_{2}}{\left(\frac{3}{8}\right)}^{b-m_{2}}\frac{b!}{m_{2}!\left(b-m_{2}\right)!}.$$

The number of constraints that are not satisfied by the sequence is $m=m_{1}+m_{2}$, which we call the mutational distance. The frequency of sequences at mutational distance m in one single frame is $P_{sing}\left(m\right)=\sum_{m_{1}=0}^{m}P(m_{1},m-m_{1})$. The frequency of exact matches is $P_{sing}\left(0\right)=q$, as above. The probability that the mutational distance is greater than or equal to *m* in any one frame is $g\left(m\right)=\sum_{m^{\prime }=m}^{s+b}P_{sing}\left(m^{\prime }\right)$. If the W frames are treated as independent, the probability that the best of these has *m* mutations is


$$P_{W}\left(m\right)=g{\left(m\right)}^{W}-g{\left(m+1\right)}^{W}.$$

For m=0, $g\left(0\right)=1$ and $g\left(1\right)=1-q$; therefore $P_{W}\left(0\right)=1-{\left(1-q\right)}^{W}\approx Wq$, as before.

**Figure 7.**
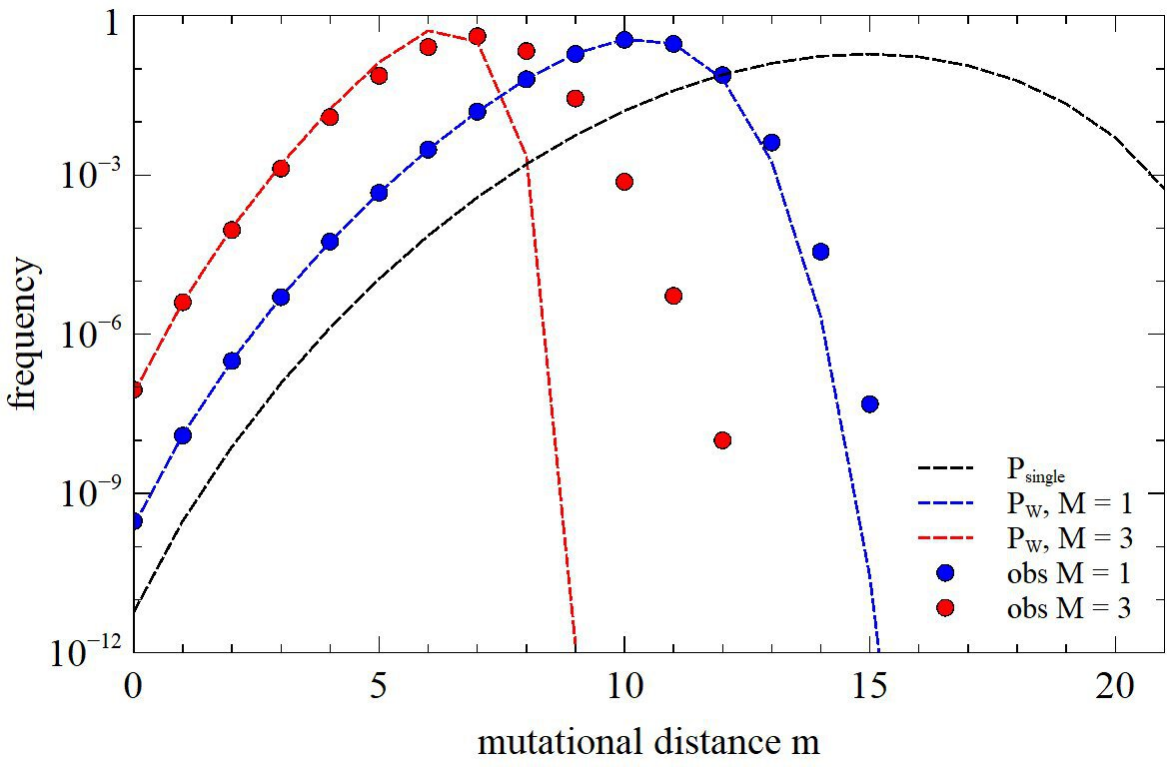
Frequency of random sequences at mutational distance m from the hammerhead secondary structure, calculated for a single frame, and for multiple frames with *M* = 1 or 3 and $L_{ran}=80$. Curves are from the theoretical formula. Data points are measured by exact analysis of random sequences.

The assumption that the frames are independent is not strictly correct because frames are overlapping. Therefore we wish to test this result by exact calculation. For the hammerhead ribozyme with $L_{ran}=80$, figure 7 shows the single frame distribution $P_{sing}(m)$ and the multiple frame distribution $P_{W}(m)$ for M=1 and M=3 (dashed curves). Data points show exact frequencies calculated from large numbers of random sequences. For M=1, we tested $10^{10}$ random sequences. For each sequence we found the smallest m from all 42 frames. For M=3, we tested $10^{8}$ random sequences and found the smallest m from all 13 244 frames. For small m, $P_{W}(m)$ is greater than $P_{sing}(m)$ by several orders of magnitude. Thus, allowing for multiple frames greatly increases the frequency of finding matching sequences. We also see that for m=1, 2 and 3, $P_{W}(m)$ is greater than $P_{W}(0)$ by several orders of magnitude. Thus, the frequency of non-exact matches is much higher than the frequency of exact matches. The data points from exact calculation are extremely close to the theory for m≤5, but differ significantly for larger *m*. The theory gives a good prediction for sequences within a few mutations. These are the sequences that we are interested in because these include the sequences that have high catalytic rate.

During an experiment, RNA sequences can be obtained by high-throughput sequencing of the DNA libraries obtained in every selection round. The resulting sequences can be clustered into groups with significant similarity, but there is little similarity between sequences in different clusters. This is what we expect if the sequences in a cluster have descended from a single copy of a ‘founder sequence’ that was initially in the random pool. If the founder sequences fold into the same structure with the same constraints, these sequences will be widely separated in sequence space because they occur in different frames. For example, the self-triphosphorylation ribozyme in figure 6c was found in three separate sequence clusters, with the same structure occurring in different frames. However, there were also many other clusters containing catalysts with different structures [25,44], and there is no reason why the sequences for the different structures should be similar.

### 2.7. Effects of beneficial mutations

As the frequency of sequences that do not match the constraints exactly is much higher than the frequency of exact matches, the founder sequences in the initial pool will usually not be at the top of fitness peaks. Also, even if a founder sequence is an exact match, it may still be possible to improve it by mutations that make compensatory changes in paired regions, or mutations that increase A without changing the conserved sites. Therefore we want to estimate the probability that a beneficial mutation which improves catalyst fitness will occur during an experiment.

The sequences recovered after each cycle are multiplied many times by PCR using DNA polymerase enzymes. The error rate of DNA polymerases such as *Taq* polymerase is around $u=1\times 10^{-5}$ per base. If there are s conserved sites and $m_{1}$ positions that do not match the conserved sequence, and if each mutation produces one of the three other bases with equal probability, then the probability that a copied sequence will have a beneficial mutation at one of the $m_{1}$ positions without having additional deleterious mutations is $p_{ben}=m_{1}(u/3){\left(1-u/3\right)}^{m_{1}-1}{\left(1-u\right)}^{s-m_{1}}\approx m_{1}u/3$. Similar formulae apply for beneficial mutations in base paired regions. The probability of a beneficial mutation in a single sequence copy is very small if the error rate is low. However, PCR is continued over many cycles, and the number of copies C produced from each original sequence increases exponentially as $C=\lambda^{n_{cyc}}$, where $n_{cyc}$ is the number of cycles of PCR in one round of selection. If there is perfect doubling, λ=2, but λ≈1.6 may be more realistic. Multiplication factors of C≈1000 are reasonable, for example $2^{10}=1024$ and $1.6^{14}=1153$.

If the frequency of sequences in cluster *i* is $p_{i}$ after n rounds, and $N_{tot}$ is the total number of sequences recovered after round n, then the number of recovered sequences in cluster *i* is ${p_{i}N}_{tot},$ and after PCR, it is ${p_{i}N}_{tot}C$. The expected number of sequences with a beneficial mutation is $N_{ben}\approx p_{i}N_{tot}Cp_{ben}$. Initially $p_{i}$ is very small, so $N_{ben}\ll 1$. However, $p_{i}$ increases exponentially over the first few rounds. We define $p_{min}=1/N_{tot}Cp_{ben}$. If $p_{i}>$$p_{min}$, then $N_{ben}>1$, and beneficial mutations will almost certainly occur. It should be remembered that $N_{tot}$ and C are very large, so $p_{min}$ may be small, even though $p_{ben}$ for a single sequence copy may be small if the DNA polymerase has a low error rate. We thus argue that after a fairly small number of rounds of selection, the frequencies of clusters of high-fitness sequences will be large enough for beneficial mutations to occur with virtual certainty.

We now consider an example to show the effects of beneficial mutations. There are 50 high fitness sequences in the initial pool, each of which founds a cluster. For simplicity, we consider just three sequences in each cluster, labelled m=0,1 and 2, corresponding to the mutational distance from the optimum structure. The catalytic rate and amplitude for sequence m in cluster i are $k_{im}$ and $A_{im}$. We choose $k_{i0}$ randomly in the range 0.5 to 1.0 for each cluster, so the maximum possible catalytic rate is $k_{cat}=1$. The amplitude $A_{i0}$ is chosen randomly in the range 0.8 to 1.0. We then assign lower rates and amplitudes for sequences 1 and 2. We set $k_{i1}=k_{i0}/Z_{1}$ and $k_{i2}=k_{i1}/Z_{2}$, where $Z_{1}$ and $Z_{2}$ are random numbers in the range 2 to 4. We set $A_{i1}=A_{i0}-a_{1}$ and $A_{i2}=A_{i1}-a_{2}$, where $a_{1}$ and $a_{2}$ are random numbers in the range 0 to 0.2.

Each cluster is founded by sequence 2 (a suboptimal sequence). The frequency of sequence 2 is $p_{i2}=10^{-14}$ initially, while $p_{i0}=p_{i1}=0$. The frequency of background sequences is $p_{back}=1-50\times 10^{-14}$. Background sequences have $k_{back}=0.001$. The frequencies of the sequences recovered after the reaction and separation steps, but before accounting for mutations during PCR, are $p_{im}^{\prime }\left(n\right)=p_{im}\left(n-1\right)R_{im}\left(\tau \right)/\bar{R}$. The frequencies after the PCR step are $p_{im}(n)$. The fraction of beneficial mutations from sequence 2 to 1 and from 1 to 0 are each set to $p_{ben}=10^{-4}.$However, mutation is only likely if the frequency of the sequence is greater than $p_{min}$, which we set as $10^{-10}$. Therefore,


$$\begin{array}{rl} p_{i0}\left(n\right) & =p_{i0}^{\prime }\left(n\right)+\varepsilon_{1}p_{i1}^{\prime }\left(n\right),\\ p_{i1}\left(n\right) & =\left(1-\varepsilon_{1}\right)p_{i1}^{\prime }\left(n\right)+\varepsilon_{2}p_{i2}^{\prime }\left(n\right),\\ p_{i2}\left(n\right) & =\left(1-\varepsilon_{2}\right)p_{i2}^{\prime }\left(n\right), \end{array}$$

where $\varepsilon_{2}=p_{ben}$ if $p_{i2}^{\prime }\left(n\right)>p_{min}$ or $\varepsilon_{2}=0$ otherwise, and $\varepsilon_{1}=p_{ben}$ if $p_{i1}^{\prime }\left(n\right)>p_{min}$ or $\varepsilon_{1}=0$ otherwise. This means that mutations from 2 to 1 take effect after a few rounds, when sequence 2 becomes frequent, and mutations from 1 to 0 take effect after several more rounds, when sequence 1 becomes frequent.

**Figure 8.**
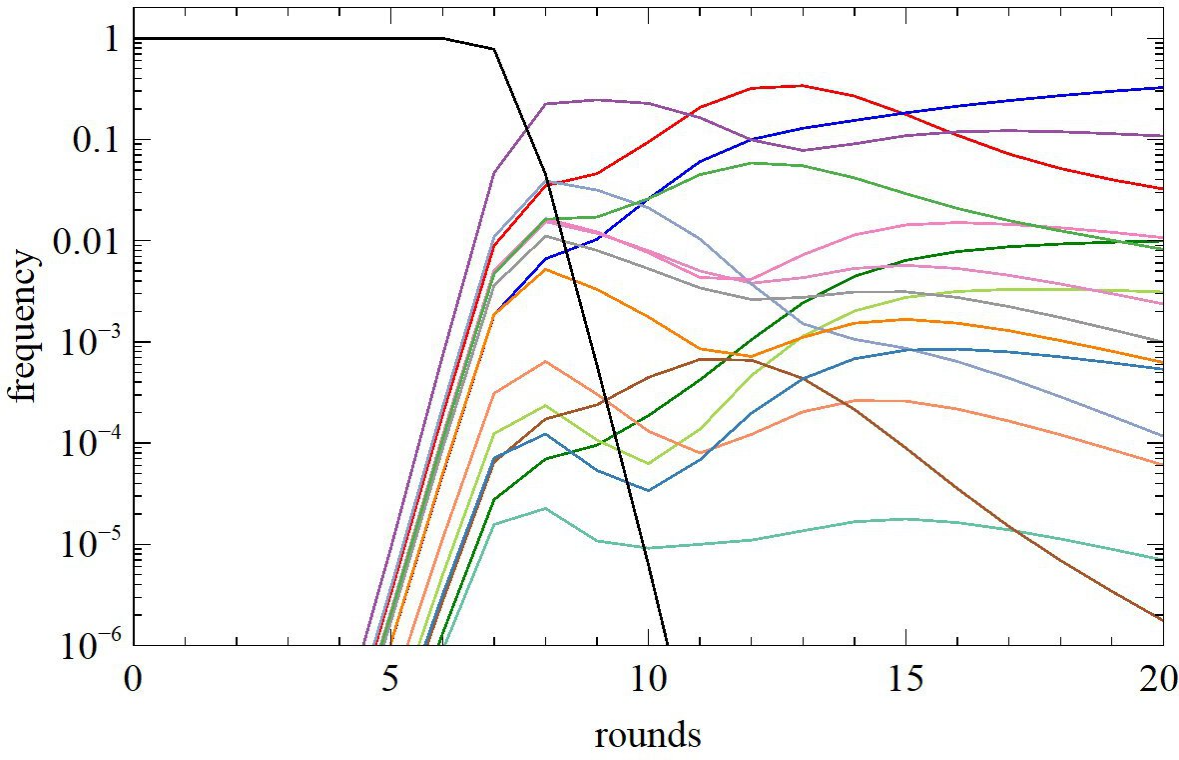
Frequency of clusters as a function of the number of selection rounds in an example where beneficial mutations occur during the experiment. There are 50 clusters in total, of which only 15 are shown.

The total frequency of sequences in the cluster is $p_{i}\left(n\right)=p_{i0}\left(n\right)+p_{i1}\left(n\right)+p_{i2}(n)$. Figure 8 shows the cluster frequencies. The reaction time is τ=5 for the first 5 rounds, and τ is decreased by a factor of 5 at each round from 6 onwards. The cluster frequencies often cross over one another, as was observed in the experimental data (figure 2). This is a sign that the relative fitnesses of clusters are changing due to the occurrence of beneficial mutations.

**Figure 9.**
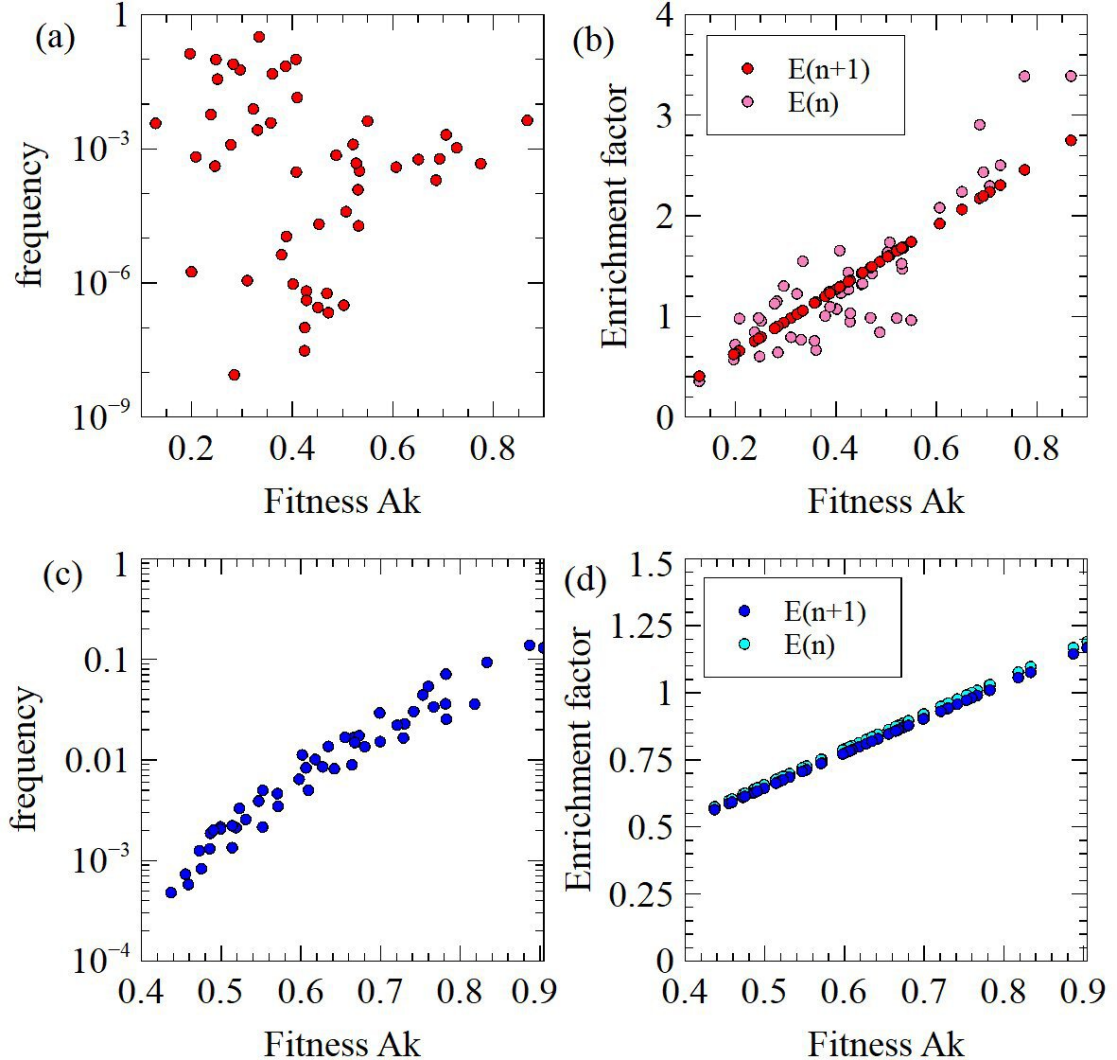
(a,b) In the 50-cluster example beginning from suboptimal sequences and allowing beneficial mutations during the experiment, the fitnesses of the clusters, $\overset{-}{Ak}$, are not correlated with the cluster frequencies, but are strongly correlated with the enrichment factors. (c,d) In the 50-cluster example beginning from optimal sequences in each cluster and allowing no beneficial mutations, the fitnesses of the clusters are strongly correlated with the cluster frequencies and with the enrichment factors.

Another feature observed in real experiments is that the frequencies of clusters correlate poorly with the fitness of the sequences, whereas the rate of increase of the cluster frequencies correlates much more strongly with the fitness [18]. This is a consequence of beneficial mutations. When τ is small, R(τ) is proportional to Ak; therefore, we refer to Ak as the fitness. The mean fitness of sequences in a cluster is $\bar{A_{i}k_{i}}=(p_{i0}(n)A_{i0}k_{i0}$ $+\;p_{i1}(n)A_{i1}k_{i1}+p_{i2}(n)A_{i2}k_{i2})/p_{i}(n)$. The rate of increase of the cluster can be measured by the enrichment factor at the current round, $E_{i}(n)=p_{i}(n)/p_{i}(n-1)$, or at the subsequent round $E_{i}(n+1)=p_{i}(n+1)/p_{i}(n)$. Figure 9 shows data from the 50-cluster example. In figure 9a,b, each cluster starts from suboptimal sequence 2, with mutations to sequences 1 and 0 occurring later and with reaction time decreasing in later rounds, as described above. Data are taken at round n=12. Figure 9a shows there is no correlation between the mean fitness of the cluster at round n and the total cluster frequency at the same round. Figure 9b shows there is a very strong correlation between the fitness and the enrichment factor at the subsequent round $E_{i}(n+1)$, and also a fairly strong correlation with the enrichment factor $E_{i}(n)$.

The very strong correlation with $E_{i}(n+1)$ occurs because the fitness of the sequences present at round n determines how much their frequency will increase during round n+1. The correlation with $E_{i}(n)$ is slightly weaker, because there was a slightly different mixture of sequences present in the cluster at the previous round. However, it is striking that there is no correlation with the cluster frequencies. This occurs because the frequency of a cluster depends on the fitness of the sequences present over the whole experiment, and not just on the sequences in the cluster at the present round.

Fitnesses of clusters change due to beneficial mutations and also due to changing the reaction time τ. We therefore compare the case with beneficial mutations with a case where there are no beneficial mutations. In figure 9c,d, we start each cluster with the optimal sequence 0 at frequency $p_{i0}=10^{-14}$, with no sequences 1 or 2 and no mutations, but with τ reducing in later rounds as before. In this case, there is strong correlation of the fitness of the cluster with the cluster frequency and also with the enrichment factors at both the current and subsequent rounds. This makes it clear that beneficial mutations produce the majority of the scatter in these plots. Hence, the primary reason for the lack of correlation between cluster frequencies and fitnesses in real data is the occurrence of beneficial mutations during the experiment.

The above treatment ignored deleterious mutations. Deleterious mutations are expected to occur more often than beneficial mutations. Nevertheless, deleterious mutations should have little effect on cluster frequencies because they will remain rare since they are depleted in each selection step. By contrast, when a beneficial mutation occurs, it gradually replaces the previous best sequence over a number of rounds, which changes the fitness of the cluster.

## 3. Discussion

We hope that the points discussed in this article will be of use to those planning new experiments. We have used rather simple mathematical models that clearly illustrate the essential points. We hope that this quantitative treatment will be useful to experimenters setting up such experiments by making clear which factors are important in which situations, and by carefully considering how the chances of success can be maximized and the possible ways in which the failure of an experiment can be avoided.

We have emphasized that, even though the total number of sequences that we begin with may be very large, the number of copies of functional sequences in the initial pool may be small. This means that stochastic effects are relevant in the early selection rounds. Even the best catalysts are not guaranteed to be recovered, because the amplitude factor A and the recovery probability Q may be less than 1. Hence, a large ratio of transcribed RNAs to DNA library size is required in order to give confidence that a functional sequence in the initial library will pass successfully through the first round. We estimated $N_{RNA}/N_{DNA}\approx 30,$ assuming QA=0.1. This means a large quantity of sequences is required in the first round, which may require multiple experimental batches.

After the first selection round is completed, functional sequences are typically present in multiple copies. From then on, the enrichment factors calculated from deterministic arguments give a good estimate of how fast functional sequences will increase. If the fitnesses of sequences remain constant over selection rounds, the cluster frequency curves never cross. However, it is likely that beneficial mutations occur during the experiment, which changes the fitnesses of clusters, and leads to crossing of the cluster frequencies. For this reason, there is a lack of correlation between the cluster fitnesses and cluster frequences, while the cluster fitness still remains correlated with the enrichment factor.

Although the initial frequency of functional sequences is very small, it is not hopelessly small. We gave many experimental examples in the introduction where functional ribozymes were successfully isolated. The isolation of ribozymes with a complex structure is facilitated because there are multiple frames in which the structure may occur (W≫1) and because suboptimal sequences are much more frequent than exact matches ($P_{W}\left(m\right)\gg P_{W}(0)$). Both these factors increase the expected frequency of functional sequences by several orders of magnitude.

The model of sequence and structure constraints used in figure 7 and table 2 is rather simplified, as it does not consider a detailed thermodynamic model of RNA folding, but the basic features of this model are confirmed by the analysis of epistatic interactions among mutations in ribozymes with conserved structures [42]. It is also well known that biological RNAs such as tRNAs and rRNAs evolve over long periods of time via compensatory changes in base-paired regions that keep the secondary structure conserved. Evolutionary models that describe compensatory substitutions in conserved RNAs have been developed for use in phylogenetic inference [47,48]. It is likely that the sequences emerging from *in vitro* selection experiments would continue to gradually evolve while maintaining the same structure if the experiment was continued for very large numbers of selection rounds.

## Data Availability

Data for figures 1 and 2 and code used to generate the other figures are available at Zenodo [49].
